# Diabetic condition induces hypertrophy and vacuolization in glomerular parietal epithelial cells

**DOI:** 10.1038/s41598-021-81027-8

**Published:** 2021-01-15

**Authors:** Takahisa Kawaguchi, Kazuhiro Hasegawa, Itaru Yasuda, Hirokazu Muraoka, Hiroyuki Umino, Hirobumi Tokuyama, Akinori Hashiguchi, Shu Wakino, Hiroshi Itoh

**Affiliations:** 1 Department of Internal Medicine, School of Medicine, Keio University, 35 Shinanomachi, Shinjuku-ku, Tokyo, 160-8582 Japan; 2 Department of Pathology, School of Medicine, Keio University, Tokyo, 160-8582 Japan

**Keywords:** Diseases, Molecular medicine, Nephrology, Pathogenesis

## Abstract

Diabetic nephropathy (DN) is accompanied by characteristic changes in the glomerulus, but little is known about the effect of diabetes on parietal epithelial cells (PECs). In this study, a descriptive analysis of PECs was undertaken in diabetic *db/db* mice and in diabetic patients. PEC hypertrophy was significantly more prominent in diabetic mice than in nondiabetic mice, and this was evident even at the early stage. Additionally, the number of vacuoles in PECs was markedly increased in diabetic mice, suggesting the presence of cellular injury in PECs in DN. Although rare, binuclear cells were observed in mice with early diabetes. In cultured PECs, a high glucose condition, compared with normal glucose condition, induced cellular hypertrophy and apoptosis. Flow cytometry showed that some PECs in the G0 phase reentered the cell cycle but got arrested in the S phase. Finally, in human diabetic subjects, hypertrophy and vacuolization were observed in the PECs. Our data showed that PECs undergo substantial changes in DN and may participate in rearrangement for differentiation into podocytes.

## Introduction

Diabetic nephropathy (DN) has been the leading cause for the initiation of renal replacement therapy, accounting for approximately one-third of cases worldwide^1^. The renal pathological changes in early DN is characterized by glomerular basement membrane (GBM) thickening and mesangial expansion, and those in late DN is characterized by glomerular nodular lesions^2,3^. These pathological alterations were mainly examined in the glomerular lesions, with focus on podocytes, mesangial cells, and endothelial cells.

In addition to the mentioned glomerular cells, parietal epithelial cells (PECs) comprise another type of resident glomerular cells; they are epithelial cells lining the Bowman’s capsule^4^. Recently, PECs have been receiving great attention as progenitor cells of podocytes^5,6^ and as barrier-forming cells that prevent periglomerular ultrafiltrate leak^7^. Although PECs were reported to be strongly involved in the pathogenesis of crescentic glomerulonephritis^8,9^ and focal segmental glomerulonephritis (FSGS)^8,10,11^, only a couple of reports have investigated the role of PECs in the pathogenesis of DN. The thickening of the Bowman’s capsule basement membrane (BBM) and capsular drop lesions have been known to be the representative morphological changes in PECs and Bowman’s capsule in DN^12,13^. Similar to the GBM, the BBM is also thickened in DN. PECs contribute to this phenomenon through the expression and secretion of Bowman’s capsule proteins, including collagen^12^. Capsular drop lesions are insudative lesions that can be identified as a round eosinophilic accumulation of materials between the PECs and the BBM in DN^13^. In human renal biopsies of DN patients, cells marking as podocytes on Bowman’s capsule increased, and Ki-67-expressing cells were identified on the glomerular tuft and Bowman’s capsule^14^. Although crescent formation is rarely observed in DN, these crescentic cells coexpressed PEC and podocyte markers, which suggested that PEC may transdifferentiate into podocytes in response to severe glomerular injury in DN^15^.

In the present study, we have examined the histopathologic changes in PECs that were evident in DN. Our results indicated that PECs were hypertrophied and injured in DN, which may be associated with the pathogenesis of DN in mice and humans.

## Results

### PEC hypertrophy and damage in DN mice

**Figure 1:**
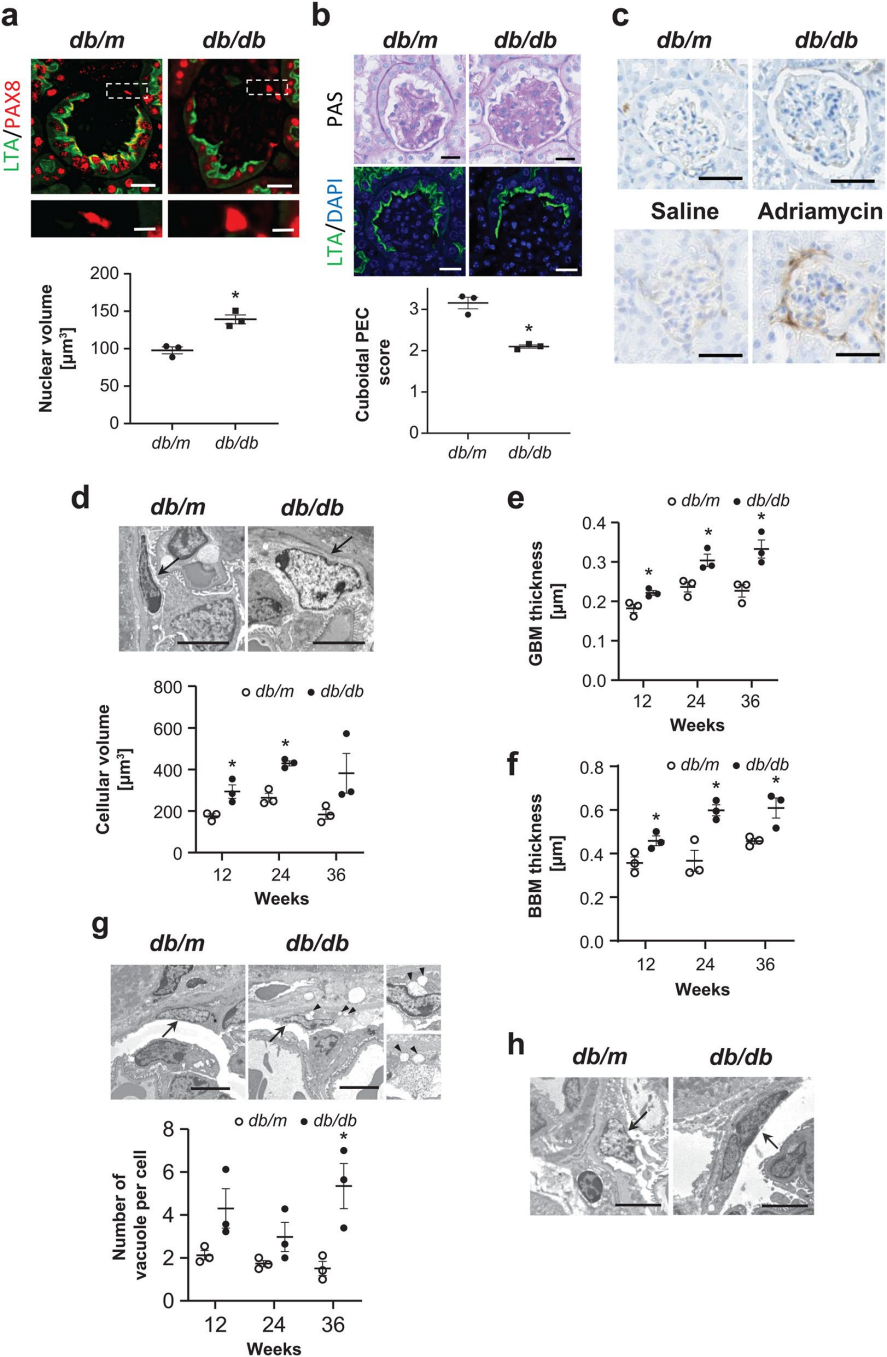
Morphological changes in PECs in diabetic *db/db* mouse. **(a)** Representative images of PAX8-LTA costaining in the 12-week-old *db/m* and *db/db* mice. The magnified view of the boxed region in the upper panel is shown in the lower panel. The dot plot graph shows the mean nuclear volume of PECs. **(b)** Representative images of PAS staining (upper) and LTA staining (lower) in the 12-week-old *db/m* and *db/db* mice. The dot plot graph shows the average of semiquantitative cuboidal PEC score in the *db/m* and *db/db* mice. **(c)** Immunostaining for CD44 in kidneys from the experimental mice groups (i.e., *db/m*, *db/db*, saline-treated, and adriamycin-treated). **(d)** Representative PECs (arrows) on transmission electron microscopy at 12 weeks of age in each mouse group. The dot plot graphs show the temporal changes in the cellular volume of PECs. **(e,f)** Temporal changes in the GBM thickness **(e)** and BBM thickness **(f)** in the *db/m* and *db/db* mice. **(g)** Representative PECs (arrows) on transmission electron microscopy at 24 weeks of age in the *db/m* and *db/db* mice. Arrowheads indicate the vacuole in PECs. The right inserts are the views on higher magnification. Quantitative analysis of the number of vacuoles per cell is shown in the lower panel. **(h)** A binucleated PEC is identified in *db/db* mice at 12 weeks of age but not in *db/m* mice. Arrows indicate PECs. PECs, parietal epithelial cells. *P < 0.05 vs. *db/m* mice. Scale bars: (**a** up) 20 μm, (a low) 5 μm, (**b**,**c**) 20 μm and (**d**,**g**,**h**) 5 μm.

Compared with nondiabetic *db/m* mice, the eight week-old diabetic *db/db* mice had significantly higher blood glucose levels (446 ± 138.3 mg/dL vs. 117 ± 7.4 mg/dL, P < 0.05), which remained persistently elevated above 600 mg/dL at 12, 24, and 36 weeks of age. The *db/db* mice developed albuminuria with increased albumin-to-creatinine ratio at as early as eight weeks of age (138.3 ± 22.4 μg/mg vs. 51.6 ± 7.4 μg/mg, P < 0.05) and remained thereafter until 36 weeks of age. Serum creatinine levels were not significantly different at 12, 24 and 36 weeks of age. In morphological analysis, light microscopy revealed nuclear hypertrophy of flat PECs in 12-week-old diabetic *db/db* mice by measuring the PAX8 positive area per cell (Fig. 1a). On the basis of the cuboidal PEC score, as described in the Methods section, the cuboidal cells on the Bowman’s capsule were significantly less prominent in 12-week-old male *db/db* mice than in male *db/m* mice, respectively (Fig. 1b). CD44, which is an activated PEC marker, was not positive in the hypertrophied PECs in the diabetic *db/db* mice at 12, 24 and 36 weeks, whereas CD44 was positive in PECs in adriamycin nephropathy, as previously reported^16^ (Fig. 1c). On electron microscopy, we confirmed PEC hypertrophy (Fig. 1d), which was evident as early as 12 weeks. Examination for the other early histological changes in DN revealed evident thickening of both the GBM and BBM from 12 weeks (Fig. 1e,f, respectively). Cellular hypertrophy sometimes induces cell apoptosis or cell damages in various cell types^17–20^. The PECs had no apparent changes in apoptosis but exhibited vacuolar degeneration, which is reported to be a result of cell damage^21,22^; time course analysis revealed that this change was evident at 36 weeks of age (Fig. 1g). In addition, we observed PECs with binuclear cells in the 12-week-old *db/db* mice (Fig. 1h). This morphological change may indicate the state of mitotic catastrophe, as previously reported in podocytes^23–25^.

### High glucose exposure induced PEC hypertrophy in vitro

**Figure 2:**
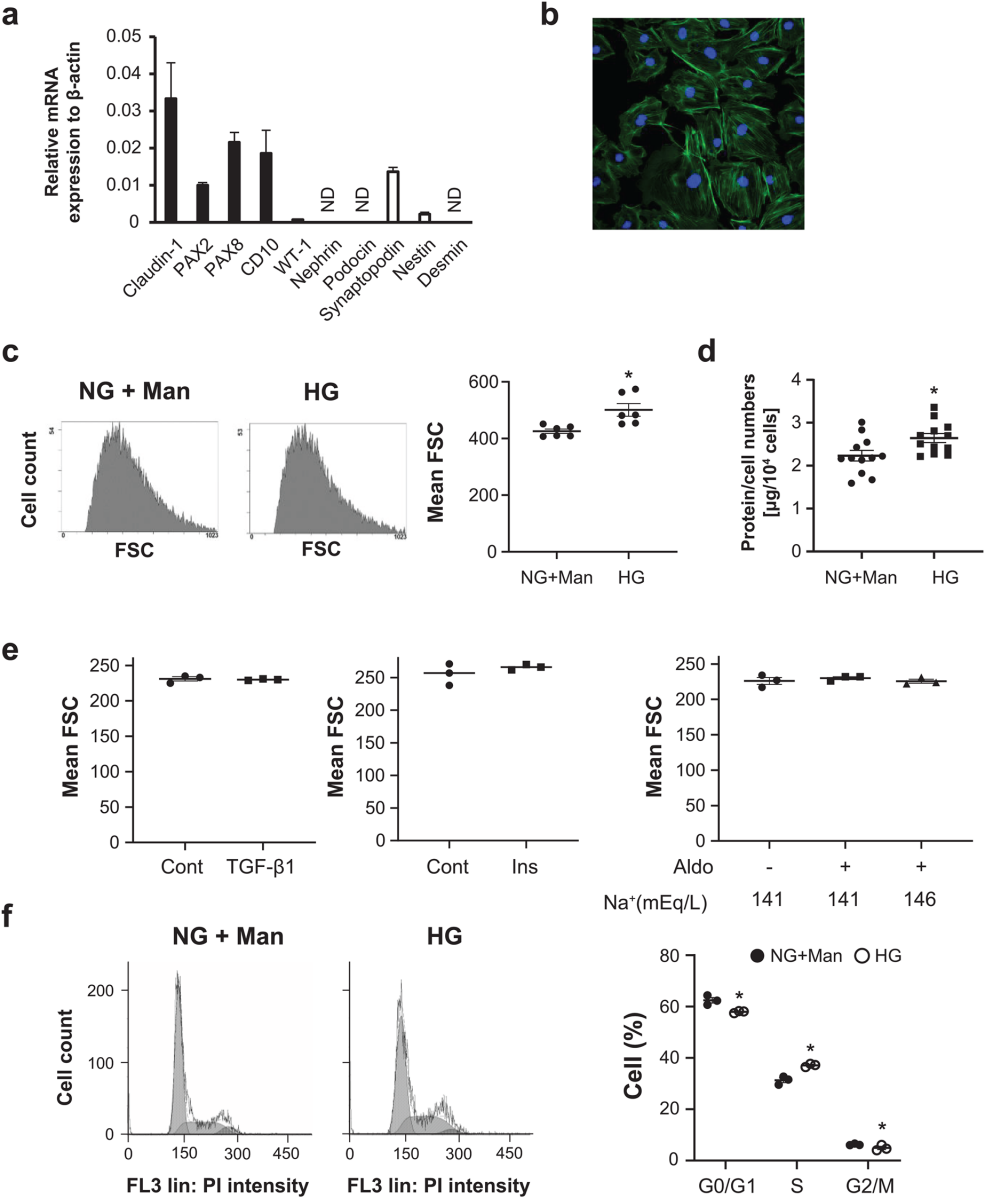
The mechanisms for PEC hypertrophy. **(a)** The relative mRNA expressions of PEC-specific (black bars) and podocyte-specific (white bars) transcripts in mouse PECs. Quantitative PCR analysis was performed in three independent experiments per each molecule. Values were normalized to the mRNA expression of β-actin as the housekeeping gene and were expressed as mean ± SE (n = 3). **(b)** A representative photograph of F-actin staining of mouse PECs. **(c)** Representative flow cytometry images of cultured PECs exposed to RPMI medium containing 5 mM of glucose (normal glucose, NG) with 25 mM of mannitol (Man) or 30 mM of glucose (high glucose, HG) for 24 h. The dot plot graph on the right shows the mean FSC (forward scatter) values. *P < 0.05 vs. NG + Man group (n = 6). **(d)** The value of the protein/cell number of PECs exposed to NG + Man or HG. *P < 0.05 vs. NG + Man group (n = 12). **(e)** Cultured PECs were exposed to RPMI medium with or without TGF-β1 (10 ng/mL), insulin (Ins, 300 nM) or aldosterone (Aldo, 10^−7^ M), in the presence of 141 or 146 mEq/L of sodium ion (n = 3). **(f)** The effects of glucose on the cell cycle in PECs. PECs were exposed to HG (30 mM) or normal glucose (5 mM) with mannitol (25 mM) culture for 24 h. The percentages of the cells in the G_0_/G_1_, S and G_2_/M phases were determined by computer analysis. *P < 0.05 vs. NG + Man group (n = 3).

To elucidate the mechanisms for the hypertrophic changes in PECs, we used an established immortalized PEC cell line^23^. The expressions of PEC-specific mRNAs, claudin-1, pax-2, pax-8, and CD10 were high, whereas the expressions of the podocyte-specific mRNAs, WT-1, nephrin, podocin, synaptopodin, nestin and desmin were low or barely detected (Fig. 2a). To further verify the morphological features of PECs in culture, we stained the cells with the cytoskeletal protein F-actin. There was a thin linear staining with F-actin in the cytoplasm and along the cell borders, reflecting the characteristic simple cytoskeletal structures of PECs, as previously described^23^ (Fig. 2b). We examined the effect of high glucose exposure. On flow cytometry, cell size was significantly greater in the PECs cultured under high glucose (HG, 30 mM) conditions than in those under normal glucose (NG, 5 mM) plus 25 mM mannitol (Man) condition (Fig. 2c). The ratio of the total cellular protein to cell number, which is another indicator of cellular hypertrophy, revealed HG-induced hypertrophic changes in the PECs (Fig. 2d). The cellular size of PECs was not increased by simulation with other hypertrophic factors, including TGF-β1^24^, insulin, or aldosterone, with or without sodium chloride^25^ (Fig. 2e). Because hypertrophy is often accompanied by cell cycle arrest^26^, we analyzed the cell cycle distribution in PECs using flow cytometry. In an HG condition, the percentage of cells decreased in the G0/G1 phase and increased in the S phase; these results indicated that some PECs in the G0/G1 phase entered the S phase after HG stimulation. However, these cells did not further progress into the G2 or M phase, indicating that the cell cycle was arrested in these cells (Fig. 2f).

### HG exposure induced cellular apoptosis in PECs in vitro

**Figure 3:**
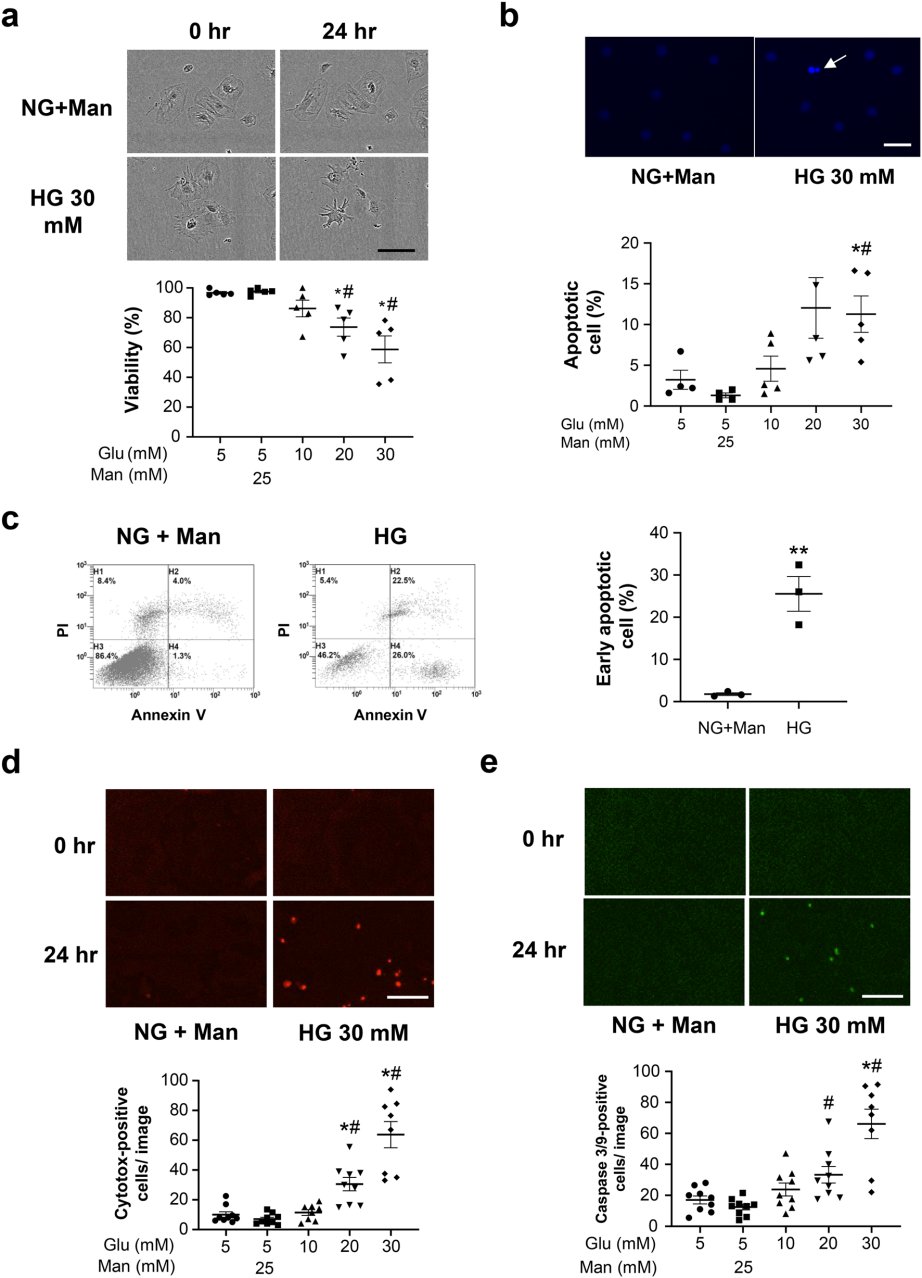
Cell damage and apoptosis of PECs in vitro. **(a)** Representative images of cultured PECs (original magnification × 40) exposed to RPMI medium containing 5 mM of glucose (normal glucose, NG) with 25 mM of mannitol (Man) or 30 mM of glucose (high glucose, HG) for 24 h. The bar graph shows quantification of the viability of the cultured PECs exposed to increasing concentrations of glucose. *P < 0.05 vs. NG, #P < 0.05 vs. NG + Man (n = 5–6). **(b)** Hoechst 33,342 staining of cultured PECs. The arrow shows a cell with condensed nuclei, which is suggestive of an apoptotic cell. The dot plot graph shows quantification of the apoptotic cultured PECs exposed to increasing concentrations of glucose. *P < 0.05 vs. NG, #P < 0.05 vs. NG + Man (n = 4–6). **(c)** Apoptosis was determined by flow cytometry. Each graph for flow cytometry represents the population of cells stained with Annexin V (X axis) and PI (Y axis). The dot plot graph shows the percentage of early apoptotic cells. **P < 0.01 vs. NG + Man (n = 3). **(d, e)** Cell viability and apoptosis were assessed using an IncuCyte imaging system. Representative microscopy images of the degree of viability, which was measured as Cytotox fluorescence (red) **(d)**, and the degree of apoptosis, which was measured as Caspase 3/7 activation (green) **(e)** at the 0 and 24 h time points. *P < 0.05 vs. NG, #P < 0.05 vs. NG + Man (n = 8–10). Scale bars: (**a**,**d**,**e**) 100 μm and (**b**) 50 μm.

Treatment of PECs with increasing concentrations of glucose for 24 h caused dose-dependent decreases in cell viability (Fig. 3a). Hoechst staining (Fig. 3b) and flow cytometry (Fig. 3c) revealed that treatment with increased concentration of glucose for 24 h increased apoptosis. Moreover, the live cell analysis system showed concentration-dependent increase in cell death (Fig. 3d) and apoptosis (Fig. 3e).

### Hypertrophy and vacuolization in human PECs in DN

**Figure 4:**
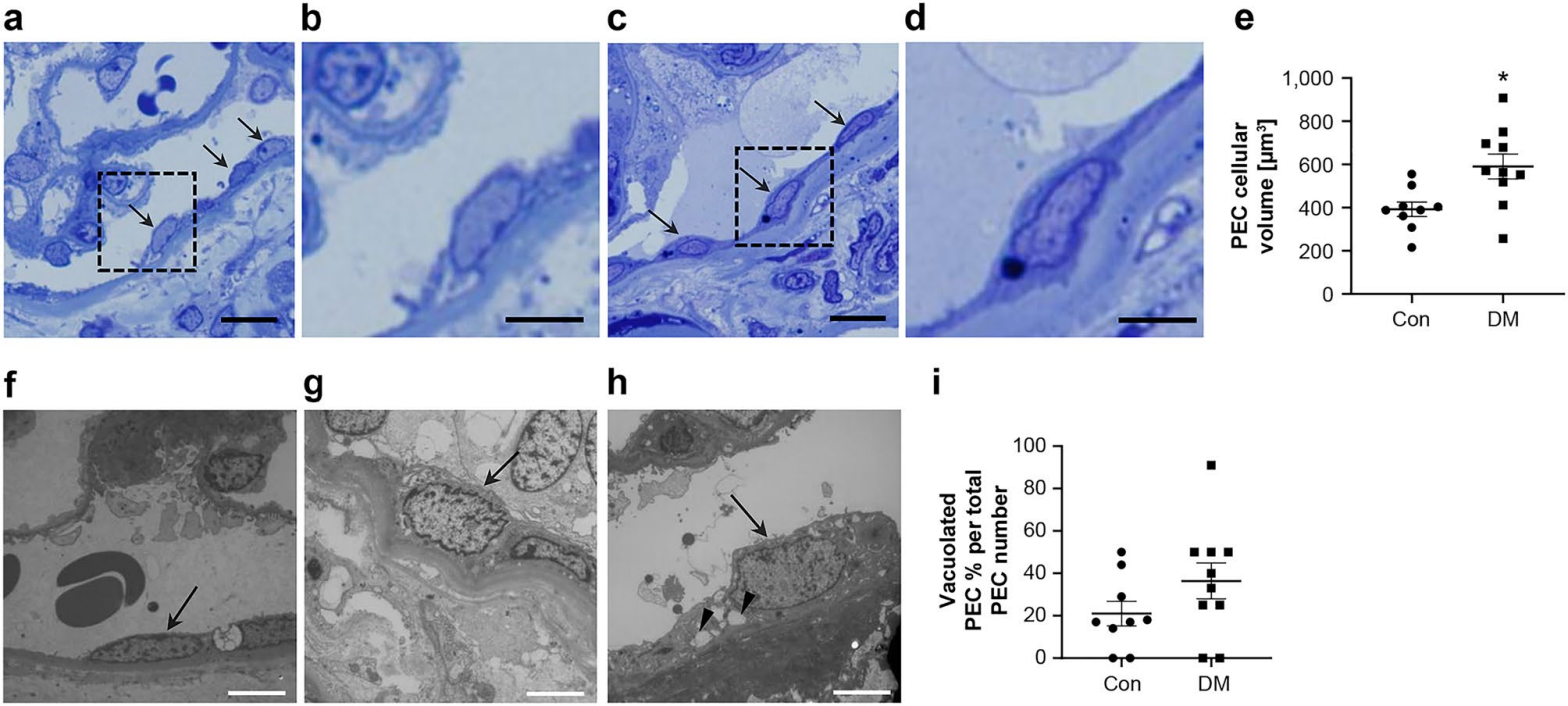
Morphological findings of PECs in diabetic human subjects. **(a–d)** PECs in human renal biopsy specimens from control patients (Con) **(a,b)** and patients with diabetes mellitus (DM) **(c,d)** (plastic-embedded semi-thin section stained with toluidine blue). Arrows indicate PECs. **(b,d)** The magnified view of the boxed region in **(a)** and **(c)**, respectively. **(e)** Quantification of PECs’ cellular volume in Con and patients with DM. **(f–h)** Transmission electron microscopic evaluation of the PECs in Con without diabetes **(f)** and patients with DM **(g,h)**. **(f)** An example of a PEC (arrow) is shown to have a flat cell body and a flat nucleus on the BBM in a nondiabetic subject. **(g,h)** The PECs in patients with DM were enlarged (arrow) and exhibited cytoplasmic vacuolization (arrowhead). Each figure was obtained from corresponding patients in Table 1, (**e**) CO-7, (**f**) DM-1, (**g**) DM-5. **(i)** Quantification of the ratio of vacuolated PEC number per total PEC number in Con and in patients with DM. Scale Bars: (**a**,**c**) 10 µm, (**b**,**d**,**f**–**h**) 5 µm.

Hypertrophied and vacuolated PECs under diabetic conditions were further examined using kidney tissue samples from patients with and without diabetes (Table 1). Quantitative analysis using plastic-embedded semi-thin sections stained with toluidine blue showed that the cellular volume of PECs was significantly increased in the diabetes group compared with the control group (p < 0.05) (Fig. 4a–e). With the help of electron microscopy, we examined the morphological changes in PECs on human renal biopsy specimens of control patients and patients with diabetes. We observed flat cell bodies and a little vacuolar changes in the control patients (Fig. 4f), whereas cellular enlargement and vacuolar changes in the patients with diabetes (Fig. 4g,h). Quantitative analysis showed that the ratio of vacuolated PEC per total PEC number in DN was high compared with that in control subjects but did not reach significance (Fig. 4i).

**Table 1:** Clinical and pathological data of controls and patients with diabetes mellitus.

Sample name	Sex	Age [years]	Serum creatinine [mg/dL]	eGFR (mL/min/1.73 m^2^)	Proteinuria [g/day]	HbA1c [%]	RPS DN Class	PEC volume [µm^3^]	Vacuolated PEC% per total PEC number
**Controls**									
CO-1	Male	22	0.94	85	0.17	4.9		504	18
CO-2	Male	20	0.80	98	0.23	5.1		404	14
CO-3	Male	51	0.83	77	0.12	5.7		308	0
CO-4	Female	39	0.65	80	0.09	5.2		215	44
CO-5	Female	39	0.72	74	0.46	5.0		388	0
CO-6	Female	30	0.63	90	0.06	5.4		406	29
CO-7	Female	65	0.75	59	0.06	5.8		361	17
CO-8	Male	55	1.16	52	0.32	6.2		555	50
**Diabetes mellitus**									
DM-1	Male	51	2.60	22	5.00	5.9	2	750	50
DM-2	Female	57	0.50	96	0.40	8.9	2	518	40
DM-3	Female	41	1.27	38	8.72	6.0	3	571	0
DM-4	Male	67	1.36	41	0.44	5.7	2	256	50
DM-5	Male	44	1.09	60	1.24	7.7	2	908	91
DM-6	Female	41	1.53	32	5.13	6.5	3	563	0
DM-7	Male	66	0.90	65	3.03	7.1	2	679	25

*CO* control, *DM* diabetes mellitus, *RPS DN class* scores as defined by the Renal Pathology Society Diabetic Nephropathy Classification.

## Discussion

In this study, we focused on the morphological alterations in PEC, which is one of the glomerular cells, in DN. We found hypertrophy of PECs and decrease in cuboidal PECs in diabetic mice. We analyzed the morphological changes on HG stimulation causing cell cycle arrest and cellular hypertrophy. Moreover, in mouse models with diabetes, HG stimulation caused PEC injury, which was represented by vacuolization. Although the pathologic implications remain to be clarified, our study identified several histologic characteristics of PECs in DN and may provide some information for further investigation on PECs.

In human DN, PEC hypertrophy was described as enlargement of the nuclear diameter and the presence of euchromatic nuclei on transmission electron microscopy, although the timing of occurrence was not clarified and the detailed mechanism was not elucidated^12^. On electron microscopy, PEC hypertrophy was observed in the early stage in the mice model of DN. PEC hypertrophy was apparent as early as the occurrence of BBM thickening; this suggested that PEC hypertrophy plays an important role in the hyperproduction of the BBM by PECs. In renal cells, HG has been reported to induce cellular hypertrophy in podocytes^17,18,27–29^, mesangial cells^30–32^ and renal tubular cells^33,34^. We tested various stimuli, including HG, insulin, TGF-β1 and aldosterone and demonstrated that only HG caused hypertrophy of the PECs. The hypertrophic changes in podocytes have been reported to be involved with the cell cycle^35^. Consistently, our cell cycle analysis suggested reentry into the cell cycle and the S phase arrest of hypertrophied PECs.

In the process of searching for the hypertrophied PECs in diabetic mice, we carefully distinguished them from activated PECs and cuboidal PECs. In activated PECs, various morphological changes were reported, including cell enlargement, nuclear swelling, structural changes from flat to cuboidal shape, and extracellular matrix production^10,36^. CD44 is a marker of activated PEC^4,9^, and the expression is higher in crescentic glomerulonephritis and in FSGS^16,37,38^. The relationship between CD44 expression and hypertrophy was suggested in DN^12,39^. Indeed, the expression of CD44 on PEC was found in patients with DN, especially in the late phase^12^. However, our findings showed hypertrophy of PECs without CD44 expression in the early stage of diabetic model mice, suggesting that PEC hypertrophy in the early stage of DN was independent of CD44 activation. The CD 44 expression in human samples with DN in the early stage needs to be further investigated. Cuboidal PECs have been defined by their shape, localization on the Bowman’s capsule and the presence of brush borders and abundant mitochondria, similar to the shape of proximal tubular cells. These cells were recently reported to be more easily activated compared with classical flat PECs in disease conditions^11^. We found that the area lined with cuboidal PECs was decreased in diabetic mice, implying that the hypertrophied PECs may extrude cuboidal PECs away into the proximal tubular area. Sex hormones^40–42^, age^43,44^ and differences in mouse strain^40^ have been described to affect the distribution of cuboidal PECs, although the factor that reduces the cuboidal PECs in diabetic mice remain to be clarified.

We demonstrated that HG exposure led to PEC apoptosis in a dose-dependent manner in vitro. PEC apoptosis has been reported in high fat feeding mice^45^, albumin overload model mice and rats^46^, transgenic mouse model of human immunodeficiency virus-associated nephropathy^47^ and mice with electrocoagulation of the renal cortex^48^ in vivo*.* Moreover, apoptosis has been reported in cultured PECs exposed to albumin^46^ and IFN-β exposure^49^. On transmission electron microscopy, our results did not show any apoptotic PECs, but there was vacuolization of PECs in early diabetes mice model and in diabetic human subjects. Because vacuolization is one of the characteristics of cell injury in podocytes^21,22^, this result suggested the presence of PEC injury in DN. In addition, the presence of binuclear PECs in diabetic mice suggested that PECs in DN undergo mitotic catastrophe instead of apoptosis. Mitotic catastrophe is cell death due to aberrant mitosis, and this implies PEC detachment from the BBM and death, as previously described in podocytes^50–52^.

Pathologically, this PEC injury was speculated to reduce glomerular regeneration in DN, because, recently, the function of PECs as progenitors of podocytes has been noticed^53^. PEC injury may also lead to periglomerular inflammation through cytokine secretion^54^, which can result in periglomerular fibrosis, as observed previously in diabetic model mice^55^ and human diabetic patients^56^.

We acknowledge some limitations in this study. First, the number of human subjects for the assessments of PEC morphology was small. Further studies including larger populations of patients with DM are necessary. Second, our examination on human diabetic sample was performed only at the late stage of DN, which is associated with profound proteinuria and elevated creatinine. Generally, there are few chances of performing renal biopsy of patients with early stage of DN. However, hypertrophy and vacuolization of PECs was observed even at late stage of DN. Further studies on human subjects with only microalbuminuria and early histologic changes are needed.

In conclusion, diabetic condition induced hypertrophy and injury in PECs. Because PECs are considered progenitors of podocytes, this injury to PECs might impair glomerular regeneration or periglomerular inflammation in a later stage. Further studies are warranted to elucidate the potential pathologic role of these morphological changes in PECs in DN.

## Methods

### Animal experiments

Obese-type diabetic *db/db* mice were used as the DN model. Two congenic strains, male *db/db* mice with diabetes (BKS.Cg-*Lepr*^*db*^*/Lepr*^*db*^) and *db/m* mice without diabetes (BKS.Cg-*Lepr*^*db*^*/*^+^), were obtained from CLEA Japan (Tokyo, Japan). Adriamycin nephropathy was induced in eight week-old male BALB/c mice (CLEA Japan) by tail vein injection of adriamycin (doxorubicin hydrochloride; Sigma-Aldrich, St. Louis, MO, USA) at a dose of 10 mg/kg body weight, as reported previously^57^. Throughout this study, the mice were housed in individual cages and were given water ad libitum. The animal room was maintained under controlled conditions (20 °C, 65% humidity and a 12 h light/12 h dark photoperiod with lights on at 8:00 a.m.). Urine albumin was determined using an Albuwell M ELISA kit (Exocell, Philadelphia, PA, USA). Urine creatinine was determined using a colorimetric microplate assay (BioAssay Systems, Hayward, CA, USA). At 12, 24 and 36 weeks of age, the mice were anesthetized by pentobarbital injection (50 mg/kg) and exsanguinated through a cervical artery incision under anesthesia. All procedures were conducted in accordance with relevant guidelines and regulations approved by the Keio University Animal Care and Use Committees. Every effort was made to minimize suffering of the mice.

### Assessment of cultured PECs hypertrophy

A conditionally immortalized mouse PEC line^26^, which was kindly provided by Professor Stuart Shankland (University of Washington, Seattle, WA, USA), was used for the cell culture studies. Differentiated PECs were stimulated with normal glucose (5 mM); normal glucose with mannitol (25 mM); HG (30 mM); 10 ng/mL of recombinant human TGF-β1 (R&D Systems, Minneapolis, MN); 300 nM of insulin (Sigma-Aldrich) or 10^−7^ M of aldosterone (Sigma-Aldrich) for 24 h. A medium with high salt content (146 mEq/L of sodium chloride; Wako, Osaka, Japan) was made, as previously reported^25^. Hypertrophy of the cultured PECs was assessed by measuring the cellular protein/cell counts and by flow cytometry, as described previously^29^. To directly determine the cell size, the cells were stained with propidium iodide (PI) and analyzed by forward light scattering using a flow cytometer (Gallios; Beckman Coulter, Brea, CA). We counted 5,000–15,000 cells per each experiment. Data were analyzed with Kaluza software (Beckman Coulter).

### Cell cycle analysis

The cell cycle status was analyzed by PI staining and flow cytometry, as previously described^58^. Briefly, 10^6^ cells were washed twice with PBS. The adherent cells were collected and fixed in ethanol overnight at 4 °C and then incubated with a mixture of PI (50 mg/mL, Sigma-Aldrich) and RNase A (25 mg/mL, Sigma-Aldrich) at 37 °C for 30 min. The level of PI fluorescence was measured with a Gallios flow cytometer, and the proportion of cells in the G_0_/G_1_, S and G_2_/M phases was measured by computer analysis. We counted 5,000–15,000 cells per each experiment.

### Apoptosis assessment

Apoptosis was measured by Hoechst 33,342 staining (Invitrogen, Thermo Fisher Scientific, Waltham, MA) and by careful morphological analysis, as previously reported^17^. The Annexin V FITC Apoptosis Detection Kit (BD Biosciences, Franklin Lakes, NJ, USA) was also used to detect apoptosis by Gallios flow cytometer. About 10^6^ cells were washed twice with PBS. The adherent cells were detached with 0.05% trypsin–EDTA after about 1 min. Annexin V FITC, in combination with PI, was used for quantitative determination of the percentage of cells undergoing apoptosis, with the aid of Kaluza software. We counted 3,000–20,000 cells. Apoptosis was also analyzed by the IncuCyte Live Cell Imaging system (Essen BioScience, Ann Arbor, MI, USA). Caspase-3/7 green stain (1:1000 dilution, Essen Biosciences) and Cytotox red stain (1:4000 dilution, Essen Biosciences) were used according to the manufacturer's instructions to label apoptotic and dead cells, respectively.

### Immunofluorescence staining of F-actin in cultured PECs

Cells were fixed with 4% paraformaldehyde for 15 min at 37 °C and were permeabilized with 0.3% Triton X-100 (Sigma-Aldrich) for 5 min at room temperature. Nonspecific binding was quenched with a blocking solution (10% normal goat serum) for 30 min at room temperature. For F-actin detection, cells were incubated with Alexa Flour 488 phalloidin (Invitrogen) after permeabilization. The sections were examined under a confocal fluorescence microscope (Carl Zeiss LSM-710; Carl Zeiss, Jena, Germany).

### RNA isolation, reverse transcription and quantitative polymerase chain reaction

The total RNA from the cultured cells was extracted using the RNeasy Plus Mini Kit (QIAGEN, Hilden, Germany). Real-time reverse transcriptase polymerase chain reaction (RT-PCR) was performed using a sequence detector (ABI Step One Plus; Applied Biosystems, Thermo Fisher Scientific, Waltham, MA) and the SYBR GREEN System (Applied Biosystems)^59^. The relative mRNA level for each gene was normalized to the mRNA express level of β-actin. The primer sequences for the forward and reverse primers are listed in Table 2.

**Table 2:** List of primer sequences used for the RT-PCR analysis in this study.

Genes	Forward primer	Reverse primer
*Claudin 1*	AGCTGTGCATGGCCTCTTGTT	AGGGCCTTTCCTATGGATGAGAGT
*PAX-2*	AGCTACATGCCCACTAAAGCACAG	CACGGGACCATGTTCGTCA
*PAX-8*	TGCTATCGCAGGCATGGTG	ACTACAGATGGTCAAAGGCTGTGG
*CD10*	TTATGTCCTGTCATAGCAGCCAAAG	CAGCAGGAATCTAGGCACCAGAG
*WT-1*	TGAAGACCCACACCAGGACTC	TGTGATGGCGGACCAATTC
*Nephrin*	GTGATGACGTCACAGAAGCAAGAA	TTTGTGTGGCATACACTGTCAGG
*Podocin*	TATGGGCCCAAACATCTACACAA	CAGAGGCAGCCAGTCAGTCA
*Synaptopodin*	GCTCGAATTCCGATGCAAATAAAC	CAGGCCAC AGTGAGATGTGAAGA
*Nestin*	GAGGTGTCAAGGTCCAGGATGTC	ACACCGTCTCTAGGGCAGTTACAA
*Desmin*	TCATGCTCAGC GAGATTGAACAG	TTACCCGATGCCCAGGTGATA
*Β actin*	CATCCGTAAAGACCTCTATGCCAAC	ATGGAGCCACCGATCCACA

### Immunohistochemical analysis

Immunohistochemistry was performed, as previously described^59^. Briefly, paraffin-embedded kidney pieces were stained using rat monoclonal antibody against CD44 (1:500; BD bioscience, San Diego, California; catalog number 550538). Before incubation, the tissue sections underwent antigen retrieval in 0.01 mol/l of sodium citrate buffer (pH 6.0) at 120 °C for 20 min. Then, the sections were incubated with biotin-labeled goat anti-rat IgG (1:200; Vector Laboratories, Burlingame, CA) antibody then treated with the Vectastain Elite ABC Kit (Vector Laboratories). Sections were examined using a biological microscope (Olympus BX53; Olympus Corporation, Tokyo, Japan). Quantitative computer-assisted image analysis was performed by a blinded observer using the Image-Pro Plus software (version 5.1; Media Cybernetics, Bethesda, MD), which is an image analysis software program.

### Immunofluorescence staining

Immunofluorescence staining for PAX8 was performed on paraffin-embedded sections using rabbit polyclonal anti-PAX8 antibody (1:100; Proteintech Group, Rosemont, IL; catalog number 10336–1-AP). Alexa Fluor 594 donkey anti-rabbit IgG (Invitrogen) was used as the secondary antibody. Immunofluorescence staining for Fluorescein-labeled LTA (Vector Laboratories) was performed in a similar manner. The slides were premounted with VECTASHIELD mounting medium with DAPI (Vector Laboratories) and kept in darkness to dry. For fluorescence microscopy, all sections were stained and analyzed simultaneously to exclude artifacts due to variable decay of the fluorochrome. The sections were examined using a confocal fluorescence microscope (Carl Zeiss LSM-710) at a magnification of 400 × .

### Identification of flat PECs and cuboidal PECs

PECs comprise mainly of flat and cuboidal PECs. We discriminated between flat and cuboidal PECs using PAX8 which stain flat PECs and cuboidal PECs in Bowman’s capsule)^60^, and LTA which only stain cuboidal PECs in Bowman’s capsule^11^. Flat PECs were identified as PAX8 positive LTA negative cells internally lining the Bowman’s capsule. We measured flat PEC nuclear volume as a substitute for flat PEC cellular volume, because the ratio between nuclear and cytoplasmic size has long been observed to be maintained at a constant value^61^. The flat PECs’ nuclear area was measured as PAX8 positive area per cell, and then the nuclear volume was calculated as described in morphological analysis. Cuboidal PECs were identified as LTA positive cells in the Bowman’s capsule for immunofluorescent staining^11^. For morphometric analysis of cuboidal PECs, each glomerulus within the kidney section was ranked on a scale of zero to five, depending on the degree of cuboidal PECs. Each rank was represented as follows: 0 = no cuboidal PECs; 1 = cuboidal PECs up to one-fourth of the glomerular circumference; 2 = cuboidal PECs < 50% of the glomerular circumference; 3 = cuboidal PECs < 75% of the glomerular circumference; 4 = cuboidal PECs < 100% of the glomerular circumference and 5 = entirely cuboidal PECs without the classical flat PECs. Age^46,47^, gender^43–45^ and genetic background^43^ affect the distribution of cuboidal PECs and flat PECs; therefore, we matched age, gender, and genetic background to compare cuboidal PECs other than compared conditions. We compared cuboidal PEC score of male *db/m* mice with that of male *db/db* mice. The background of mice is shown in Supplementary Table S1. Thirty glomeruli were obtained for each sample, and the total mean score was calculated.

### Electron microscopy

The kidney tissues were fixed on 2% glutaraldehyde and embedded in epoxy resin. The semi-thin sections stained with toluidine blue were inspected under the light microscope. The ultrathin sections, stained with uranyl acetate and lead citrate, were investigated and snapped using an electron microscope. To evaluate PEC morphometry, electron micrographs of at least five glomeruli per kidney were randomly obtained from each mouse and observed at a magnification of 2000 × . To evaluate GBM thickness and BBM thickness, a mean of 30 measurements were taken per kidney at random sites where the GBM and BBM were displayed in best cross-section as previously described^62^.

### Human renal biopsy specimens

We obtained renal needle biopsy specimens from 15 patients. We retrospectively identified eight control cases without diabetes and seven cases with diabetes. Control cases were obtained from patients without glomerular abnormalities in light microscopy. Before study enrollment, written informed consent was obtained from all patients. The study was performed in accordance with the Declaration of Helsinki, and the study protocol was approved by the human ethics review committee of the Department of Internal Medicine, School of Medicine, Keio University. Patients for whom fewer than nine PECs were observed on biopsy were excluded. PECs with sclerotic glomeruli were excluded. All visible PECs from each human sample were assessed by morphology. All the biopsies were categorized based on the pathologic classification of the Renal Pathology Society (RPS) Diabetic Nephropathy Classification^3^. PEC area was measured in plastic-embedded semi-thin sections stained with toluidine blue using light microscopy at a magnification of 1000 × . The area measurement was performed using Image-Pro Plus software. The volume was calculated as described in morphological analysis below. Vacuolated PECs were counted and assessed by the ratio to the total PEC number using electron microscopy. The patient clinical data at the time of renal biopsy and the pathological data are summarized in Table 1.

### Morphological analysis

Podocyte volume has been estimated using various methods^63–66^. However, to the best of our knowledge, a method to quantitatively estimate PEC volume and PEC nuclear volume has not been reported to date. Therefore, we substituted the measurement methods of podocytes for those of PECs. First, for counting the PEC number, we used the Weibel and Gomez method. Briefly, the PEC number was calculated from the cell density per volume (Nc_v_) and the volume density of PECs (Vc_v_) according to the equation: Nc_v_ = k/β × Nc_A_^1.5^/Vc_v_^0.5^ with β = 1.5 and k = 1. The average volumes of individual PEC and PEC nuclei were calculated with V_c_ = Vc_v_ × V_glom_^66^.

### Statistical analysis

Data are expressed as the mean ± standard error of mean and were analyzed by one-way analysis of variance, followed by the Bonferroni post hoc test. The criterion for statistical significance was a P value < 0.05.

## Supplementary Information

Supplementary Table 1.

## References

[1] Reutens AT, Atkins RC (2011). Epidemiology of diabetic nephropathy. Contrib. Nephrol..

[2] Jefferson JA, Shankland SJ, Pichler RH (2008). Proteinuria in diabetic kidney disease: A mechanistic viewpoint. Kidney Int..

[3] Tervaert TW (2010). Pathologic classification of diabetic nephropathy. J. Am. Soc. Nephrol..

[4] Shankland SJ, Smeets B, Pippin JW, Moeller MJ (2014). The emergence of the glomerular parietal epithelial cell. Nat Rev Nephrol..

[5] Kaverina NV (2019). Dual lineage tracing shows that glomerular parietal epithelial cells can transdifferentiate toward the adult podocyte fate. Kidney Int..

[6] Eng DG (2015). Glomerular parietal epithelial cells contribute to adult podocyte regeneration in experimental focal segmental glomerulosclerosis. Kidney Int..

[7] Ohse T (2009). A new function for parietal epithelial cells: A second glomerular barrier. Am. J. Physiol. Renal Physiol..

[8] Eymael J (2018). CD44 is required for the pathogenesis of experimental crescentic glomerulonephritis and collapsing focal segmental glomerulosclerosis. Kidney Int..

[9] Smeets B (2009). Tracing the origin of glomerular extracapillary lesions from parietal epithelial cells. J. Am. Soc. Nephrol..

[10] Smeets B (2011). Parietal epithelial cells participate in the formation of sclerotic lesions in focal segmental glomerulosclerosis. J. Am. Soc. Nephrol..

[11] Kuppe C (2019). Novel parietal epithelial cell subpopulations contribute to focal segmental glomerulosclerosis and glomerular tip lesions. Kidney Int..

[12] Holderied A (2015). Glomerular parietal epithelial cell activation induces collagen secretion and thickening of Bowman's capsule in diabetes. Lab. Invest..

[13] Stout LC, Kumar S, Whorton EB (1994). Insudative lesions—their pathogenesis and association with glomerular obsolescence in diabetes: A dynamic hypothesis based on single views of advancing human diabetic nephropathy. Hum. Pathol..

[14] Andeen NK (2015). The phenotypes of podocytes and parietal epithelial cells may overlap in diabetic nephropathy. Kidney Int..

[15] Gaut JP, Hoshi M, Jain S, Liapis H (2014). Claudin 1 and nephrin label cellular crescents in diabetic glomerulosclerosis. Hum. Pathol..

[16] Okamoto T (2013). Prevalence of CD44-positive glomerular parietal epithelial cells reflects podocyte injury in adriamycin nephropathy. Nephron Exp Nephrol..

[17] Lee SH (2015). Podocyte hypertrophy precedes apoptosis under experimental diabetic conditions. Apoptosis.

[18] Kim DK (2012). Translationally controlled tumor protein is associated with podocyte hypertrophy in a mouse model of type 1 diabetes. Diabetologia.

[19] Advani A (2011). Inhibition of the epidermal growth factor receptor preserves podocytes and attenuates albuminuria in experimental diabetic nephropathy. Nephrology (Carlton)..

[20] Hara Y (2011). Rho and Rho-kinase activity in adipocytes contributes to a vicious cycle in obesity that may involve mechanical stretch. Sci Signal..

[21] Ueda S (2015). ENOS deficiency causes podocyte injury with mitochondrial abnormality. Free Radic. Biol. Med..

[22] Catanuto P (2012). In vivo 17beta-estradiol treatment contributes to podocyte actin stabilization in female db/db mice. Endocrinology.

[23] Ohse T (2008). Establishment of conditionally immortalized mouse glomerular parietal epithelial cells in culture. J. Am. Soc. Nephrol..

[24] Monkawa T, Hiromura K, Wolf G, Shankland SJ (2002). The hypertrophic effect of transforming growth factor-beta is reduced in the absence of cyclin-dependent kinase-inhibitors p21 and p27. J. Am. Soc. Nephrol..

[25] Yamamuro M (2006). Direct effects of aldosterone on cardiomyocytes in the presence of normal and elevated extracellular sodium. Endocrinology.

[26] Shankland SJ, Wolf G (2000). Cell cycle regulatory proteins in renal disease: Role in hypertrophy, proliferation, and apoptosis. Am. J. Physiol. Renal Physiol..

[27] Lv W (2015). Mycophenolate mofetil inhibits hypertrophy and apoptosis of podocyte in vivo and in vitro. Int. J. Clin. Exp. Med..

[28] Romero M, Ortega A, Izquierdo A, Lopez-Luna P, Bosch RJ (2010). Parathyroid hormone-related protein induces hypertrophy in podocytes via TGF-beta(1) and p27(Kip1): Implications for diabetic nephropathy. Nephrol. Dial. Transplant..

[29] Xu ZG (2005). Angiotensin II receptor blocker inhibits p27Kip1 expression in glucose-stimulated podocytes and in diabetic glomeruli. Kidney Int..

[30] Zhuo L (2011). NAD blocks high glucose induced mesangial hypertrophy via activation of the sirtuins-AMPK-mTOR pathway. Cell. Physiol. Biochem..

[31] Abdel-Wahab N, Weston BS, Roberts T, Mason RM (2002). Connective tissue growth factor and regulation of the mesangial cell cycle: Role in cellular hypertrophy. J. Am. Soc. Nephrol..

[32] Wolf G, Schroeder R, Zahner G, Stahl RAK, Shankland SJ (2001). High glucose-induced hypertrophy of mesangial cells requires p27Kip1, an inhibitor of cyclin-dependent kinases. Am. J. Pathol..

[33] Sun L, Kondeti VK, Xie P, Raparia K, Kanwar YS (2011). Epac1-mediated, high glucose-induced renal proximal tubular cells hypertrophy via the Akt/p21 pathway. Am. J. Pathol..

[34] Huang JS, Chuang LY, Guh JY, Huang YJ, Hsu MS (2007). Antioxidants attenuate high glucose-induced hypertrophic growth in renal tubular epithelial cells. Am. J. Physiol. Renal Physiol..

[35] Li JJ (2007). Podocyte biology in diabetic nephropathy. Kidney Int. Suppl..

[36] Smeets B (2004). The parietal epithelial cell: A key player in the pathogenesis of focal segmental glomerulosclerosis in Thy-1.1 transgenic mice. J. Am. Soc. Nephrol..

[37] Fatima H (2012). Parietal epithelial cell activation marker in early recurrence of FSGS in the transplant. Clin. J. Am. Soc. Nephrol..

[38] Nakamura H, Kitazawa K, Honda H, Sugisaki T (2005). Roles of and correlation between alpha-smooth muscle actin, CD44, hyaluronic acid and osteopontin in crescent formation in human glomerulonephritis. Clin. Nephrol..

[39] Zhao X (2019). Albumin induces CD44 expression in glomerular parietal epithelial cells by activating extracellular signal-regulated kinase 1/2 pathway. J. Cell. Physiol..

[40] Yabuki A, Suzuki S, Matsumoto M, Nishinakagawa H (1999). Morphometrical analysis of sex and strain differences in the mouse nephron. J. Vet. Med. Sci..

[41] Carpino F, Barberini F, Familiari G, Melis M (1976). Columnar cells of the parietal layer of Bowman's capsule and their relationship with the sexual cycle in normal female mice. Experientia.

[42] Crabtree C (1940). Sex differences in the structure of Bowman's capsule in the mouse. Science.

[43] Tabatabai NM (2014). De novo expression of sodium-glucose cotransporter SGLT2 in Bowman's capsule coincides with replacement of parietal epithelial cell layer with proximal tubule-like epithelium. J. Membr. Biol..

[44] Haley DP, Bulger RE (1983). The aging male rat: Structure and function of the kidney. Am. J. Anat..

[45] Szeto HH (2016). Protection of mitochondria prevents high-fat diet-induced glomerulopathy and proximal tubular injury. Kidney Int..

[46] Chang AM (2012). Albumin-induced apoptosis of glomerular parietal epithelial cells is modulated by extracellular signal-regulated kinase 1/2. Nephrol. Dial. Transplant..

[47] Barisoni L, Bruggeman LA, Mundel P, D'Agati VD, Klotman PE (2000). HIV-1 induces renal epithelial dedifferentiation in a transgenic model of HIV-associated nephropathy. Kidney Int..

[48] Schulte K (2014). Origin of parietal podocytes in atubular glomeruli mapped by lineage tracing. J. Am. Soc. Nephrol..

[49] Migliorini A (2013). The antiviral cytokines IFN-alpha and IFN-beta modulate parietal epithelial cells and promote podocyte loss: Implications for IFN toxicity, viral glomerulonephritis, and glomerular regeneration. Am. J. Pathol..

[50] Liapis H, Romagnani P, Anders HJ (2013). New insights into the pathology of podocyte loss: Mitotic catastrophe. Am. J. Pathol..

[51] Tang H (2017). MDM2 is implicated in high-glucose-induced podocyte mitotic catastrophe via Notch1 signaling. J. Cell. Mol. Med..

[52] Hara M, Oohara K, Dai DF, Liapis H (2019). Mitotic catastrophe causes podocyte loss in the urine of human diabetics. Am. J. Pathol..

[53] Appel D (2009). Recruitment of podocytes from glomerular parietal epithelial cells. J. Am. Soc. Nephrol..

[54] Medzhitov R (2010). Inflammation 2010: New adventures of an old flame. Cell.

[55] Takashima S (2016). Stromal cell-derived factor-1 is upregulated by dipeptidyl peptidase-4 inhibition and has protective roles in progressive diabetic nephropathy. Kidney Int..

[56] Jenkins J, Brodsky SV, Satoskar AA, Nadasdy G, Nadasdy T (2011). The relevance of periglomerular fibrosis in the evaluation of routine needle core renal biopsies. Arch. Pathol. Lab. Med..

[57] Wang Y, Wang YP, Tay YC, Harris DC (2000). Progressive adriamycin nephropathy in mice: Sequence of histologic and immunohistochemical events. Kidney Int..

[58] Yang YL (1998). Interaction between high glucose and TGF-beta in cell cycle protein regulations in MDCK cells. J. Am. Soc. Nephrol..

[59] Hasegawa K (2013). Renal tubular Sirt1 attenuates diabetic albuminuria by epigenetically suppressing Claudin-1 overexpression in podocytes. Nat. Med..

[60] Sweetwyne MT (2017). The mitochondrial-targeted peptide, SS-31, improves glomerular architecture in mice of advanced age. Kidney Int..

[61] Chan YH, Marshall WF (2010). Scaling properties of cell and organelle size. Organogenesis.

[62] Reiniger N (2010). Deletion of the receptor for advanced glycation end products reduces glomerulosclerosis and preserves renal function in the diabetic OVE26 mouse. Diabetes.

[63] Fukuda A (2012). Angiotensin II-dependent persistent podocyte loss from destabilized glomeruli causes progression of end stage kidney disease. Kidney Int..

[64] Herbach N (2009). Diabetic kidney lesions of GIPRdn transgenic mice: Podocyte hypertrophy and thickening of the GBM precede glomerular hypertrophy and glomerulosclerosis. Am. J. Physiol. Renal Physiol..

[65] Pagtalunan ME (1997). Podocyte loss and progressive glomerular injury in type II diabetes. J. Clin. Invest..

[66] Gross M-L (2003). ACE-inhibitors but not endothelin receptor blockers prevent podocyte loss in early diabetic nephropathy. Diabetologia.

