# Supplementary material for: Diabetic condition induces hypertrophy and vacuolization in glomerular parietal epithelial cells
Source: Sci Rep. 2021 Jan 15;11:1515. doi: 10.1038/s41598-021-81027-8 (PMC7810998; doi:10.1038/s41598-021-81027-8)
Supplement: Supplementary file 1 — Supplementary Table 1. [file 41598_2021_81027_MOESM1_ESM.docx]

**Diabetic condition induces hypertrophy and vacuolization in glomerular parietal epithelial cells**

**Takahisa Kawaguchi^1^, Kazuhiro Hasegawa^1^, Itaru Yasuda^1^, Hirokazu Muraoka^1^, Hiroyuki Umino^1^, Hirobumi Tokuyama^1^, Akinori Hashiguchi^2^, Shu Wakino^1*^, Hiroshi Itoh^1^**

**^1^Department of Internal Medicine, School of Medicine, Keio University, Tokyo 160-8582, Japan**

**^2^Department of Pathology, School of Medicine, Keio University, Tokyo 160-8582, Japan**

**Corresponding author: Shu Wakino, MD**

**Department of Internal Medicine; School of Medicine, Keio University**

**35 Shinanomachi, Shinjuku-ku, Tokyo 160-8582, Japan**

**Phone: +81-3-5363-3796, Fax: +81-3-3359-2745, Email: shuwakino@z8.keio.jp**

**Supplementary Table**

**Supplementary Table 1. Background data of *db/m* mice and *db/db* mice in the analysis of cuboidal PEC score**

| mice | gender | genetic background |
| --- | --- | --- |
| *db/m* | Male | BKS.Cg-*Dock7^m^*+/+*Lepr^db^*/Jcl |
| *db/db* | Male | BKS.Cg-+*Lepr^db^*/+*Lepr^db^*/Jcl |
